# Antimicrobial efficacy of Odontopaste in endodontics: a systematic review

**DOI:** 10.1038/s41432-024-01000-y

**Published:** 2024-03-27

**Authors:** Rachael Kendell-Wall, Jennifer-Thuy Nguyen, Fauve Salleras, Amandeep Singh Kamboj, Serene Aimee Diwen Tan, Vaidehi Manish Trivedi, João Martins de Mello-Neto, Rodrigo Rodrigues Amaral

**Affiliations:** College of Medicine and Dentistry, James Cook University, Campus Smithfield, Cairns, QLD Australia

**Keywords:** Root canal treatment, Root canal treatment

## Abstract

**Aim:**

To evaluate the efficacy of Odontopaste in reducing the microbial load in endodontics compared to other intracanal medicaments.

**Materials and methods:**

The literature was electronically searched on PubMed, Google Scholar, Scopus, Ovid Medline and Web of Science. In-vitro, ex-vivo and in-vivo studies that evaluated the antimicrobial efficacy of Odontopaste were included. The risk of bias was assessed using the Quality Assessment Tool for In Vitro Studies.

**Results:**

A total of four in-vitro studies were included in the systematic review. One study showed that Odontopaste had significantly more microbial cell growth on roots in all dentine depths compared to other medicaments or test agents. Another study found that Odontopaste significantly decreased colony-forming units compared to propolis and chlorhexidine. Further results showed that Odontopaste did not significantly decrease microbial numbers when used in isolation. Additionally, combining Odontopaste and calcium hydroxide did not enhance the effectiveness of calcium hydroxide. The studies had a medium to high risk of bias.

**Conclusion:**

There is insufficient high-quality evidence to assess the antimicrobial efficacy of Odontopaste compared to other intracanal medicaments. Further research is required to determine Odontopaste’s efficacy as an antimicrobial medicament in endodontics.

Key points

- The lack of evidence regarding Odontopaste’s antimicrobial efficacy compared to other medicaments is highlighted.
- Establishing the lack of research on Odontopaste may encourage further studies with consistent methodologies, minimising the risk of bias and increasing evidence towards research-based practice.
- Odontopaste may have potential in endodontics, but further clinical and in-vitro studies are necessary to confirm its efficacy.

## Introduction

Endodontic infections are polymicrobial as they are mediated by various microbes, including fungi and bacteria^1,2^. Common genera of endodontic pathogens include *Enterococcus*, *Porphyromonas, Prevotella, Fusobacterium, Streptococcus*, *Actinomyces*, *Candida*, *Peptostreptococcus, Bacteroides and Eubacterium*^1,2^. It is well established in endodontics that microorganisms are responsible for initiating and proliferating periradicular disease^1,2^. Consequently, effective disinfection of the root canal system is one of the main goals of endodontic treatment^1,3,4^. Total microbial eradication is challenging considering the complex anatomy of root canal systems^3^; however, root canal treatment aims to reduce the microbial load within the root canal to allow for periapical healing^3,5^. Such healing can occur when the number of microbial cells is lower than the threshold that causes disease^3,6^. In primary root canal infections, the success of endodontic procedures in reducing bacterial load, considering only microbial factors, is influenced by the type of pathogens present and the duration of infection^1^. In persistent infections, the efficacy of root canal procedures can be compromised by the development of microbial resistance and biofilm formation^3^. Hence, measures such as intracanal medicaments and chemomechanical preparation are beneficial for maximum bacterial reduction^3,5^.

Currently, calcium hydroxide is the most established intracanal dressing for microbial reduction^7^. It functions by altering the cell wall of lipopolysaccharides resulting in reduced antigenicity, and simultaneously establishing a highly alkaline environment in root canal systems^7^. Besides the inhibition of bacterial growth through hard tissue barrier formation, its high alkaline properties allow for the disinfection of root canal systems by dissolving remnants of organic tissue and microorganisms^7,8^. The use of calcium hydroxide as an intracanal medication has been proven to improve the microbiological status of the root canal system and promote periradicular healing^9^. However, with the advent of antibiotics in modern dentistry, additional intracanal medicaments are available to eliminate or reduce the bacterial load in root canals^10^. Despite anti-inflammatory actions, the effectiveness and role of antibiotic-containing medicaments in endodontics require further research to ensure that current practices are evidence-based and treatment standards are updated^10,11^.

Odontopaste (Australian Dental Manufacturing, Kenmore Hills, QLD, Australia) was released in 2008 and is a zinc-oxide-based intracanal medicament consisting of 5% clindamycin hydrochloride, 1% triamcinolone acetonide, and 0.5% calcium hydroxide^12^. It is advertised as a dressing for reducing inflammation and postoperative pain and preventing bacterial growth in root canal systems^12^. Odontopaste also possesses analgesic effects in endodontic treatment and does not stain teeth, as demonstrated in other intracanal medications such as Ledermix^13–15^. In addition, clindamycin, one of the components of Odontopaste, effectively targets endodontic pathogens^11^. All these components make Odontopaste an intracanal dressing with potential benefits in endodontic therapy. However, no clinical studies prove its efficacy in the current literature.

Despite the lack of evidence, Odontopaste is widely used by many dental practitioners in endodontic treatment in Australia. A global survey of 543 endodontists and postgraduate students specialising in endodontics has shown that a subgroup comprised of 85.9% Australian-qualified endodontists utilised Odontopaste at a rate of 47.4%^16^. In contrast, prevalence rates of approximately 2% and 4.8% were observed in groups predominantly composed of USA and Britain-qualified professionals, respectively^16^. Consequently, this systematic review aims to evaluate the efficacy of Odontopaste in reducing the microbial load compared to other intracanal medications.

## Materials and methods

### Protocol and registration

This systematic review was reported following The Preferred Reporting Items for Systematic Reviews and Meta-Analyses (PRISMA) statement guidelines. The systematic review proposal was registered in PROSPERO (CRD42023418576).

### Focus question

The PICO framework was used to form the following focus question: “How does Odontopaste compare with other intracanal medicaments in reducing the microbial load in endodontics?”

- Population: human or bovine dentine slices.
- Intervention: odontopaste as an intracanal medicament in endodontics.
- Comparison: any other intracanal medicament.
- Outcome: the effectiveness of Odontopaste in reducing the microbial load in the root canal.

### Inclusion criteria

Studies that evaluated the antimicrobial effectiveness of Odontopaste as an intracanal medicament in the context of endodontics were included. It also included studies that compared the effectiveness of Odontopaste to other intracanal medicaments. Studies that used samples from dentine at any depth and at least one type of microorganism were selected. In-vitro, ex-vivo and in-vivo studies from any country were eligible for inclusion.

### Exclusion criteria

The exclusion criteria included studies not in English and studies where the full text was inaccessible. Furthermore, studies that did not evaluate antimicrobial efficacy were also excluded. Studies that did not examine Odontopaste in the context of endodontic treatment were excluded. Studies, where the only test agent was Odontopaste combined with other intracanal medicaments, were ineligible. Case reports, case series, literature reviews, editorials, surveys, guidelines and systematic reviews were also excluded.

### Information sources

Electronic searches were conducted in the following databases: PubMed, Google Scholar, Scopus, Ovid Medline and Web of Science. PubMed, Scopus, Ovid Medline and Web of Science offer a wide range of reliable, evidence-based literature on health-related topics. Even though studies vary in quality on Google Scholar, it was included to ensure any relevant studies were not missed. On August 28, 2023, the searches were conducted in each database and exported to EndNote for the study selection process. This date was chosen so the searches could be conducted close to the due date for the systematic review to ensure any new studies were included. Table 1 shows the search strategy, which was kept consistent and only adapted to suit the format of each database. Filters include the English language and full text available. Medical Subject Headings (MeSH) terms, Boolean operators (OR, AND) and keywords were used to search for eligible studies.

**Table 1:** Search truncation and keywords for each database.

Database	Search truncation and key words
PubMed	((((“endodontics”[MeSH Terms] OR “root canal therapy”[MeSH Terms] OR “root canal preparation”[MeSH Terms]) OR ((“root canal”[Title/Abstract] OR “endodont*“[Title/Abstract]) AND ((“odontopaste”[Title/Abstract] OR “intracanal medicament*“[Title/Abstract])
Google Scholar	With all of the words: odontopaste With at least one of the words: endodontic “root canal therapy” “root canal preparation” “root canal” “intracanal medicament”
Scopus	(TITLE-ABS-KEY ((endodont* OR “root canal therapy” OR “root canal preparation” OR “root canal”)) AND TITLE-ABS-KEY ((odontopaste OR “intracanal medicament*“)))
Ovid Medline	1. endodontics/or “root canal therapy”/ or “root canal preparation”/ 2. (“root canal” or endodont*) 3. 1 or 2 4. (odontopaste or “intracanal medicament*“) 5. Dentistry/ 6. 3 and 4
Web of Science	(endodont* OR “root canal therapy” OR “root canal preparation” OR “root canal”) (All Fields) AND (odontopaste OR “intracanal medicament*“) (All Fields)

### Selection process

Once all search results were exported to EndNote, duplicates were deleted. Next, two independent researchers (AK and FS) assessed the title and abstract of each article alongside the inclusion and exclusion criteria. The full text of the selected articles was exported to EndNote and evaluated by the two independent researchers. Discrepancies were resolved by the third independent researcher and verified by JMMN and RRA. Cohen’s Kappa statistic was 1 for the full-text analysis, meaning there was unanimous agreement^17^.

### Risk of bias assessment

Two researchers (ST and AK) independently assessed the risk of bias in the four included studies. Discrepancies were resolved by a third researcher (JMMN). The Quality Assessment Tool for In Vitro Studies (QUIN Tool) was used, and Table 2 shows the 12 criteria^18^. Each criterion is given two points for adequately specified, one for inadequately specified, and zero for not specified^18^. A final score was calculated for each study: (total score × 100)/(2 × number of criteria applicable)^18^. A score of >70% indicated a low risk of bias, 50–70% was classified as a medium risk of bias, and <50% was a high risk of bias^18^.

**Table 2:** QUIN Tool criteria^18^.

Criteria Number	Criteria	Description
1	Clearly stated aims/objectives.	Study should clearly state aims and/or objectives, which should then be followed throughout.
2	Detailed explanation of sample size calculation.	Details regarding method by which given sample size calculated should be clearly stated. Details regarding software programme, formula, and parameters used for calculation of sample size should also be specified.
3	Detailed explanation of sampling technique.	Details regarding predefined population from which sample has been selected. Details of sampling technique and inclusion and exclusion criteria should be clearly stated.
4	Details of comparison group.	Details of comparison group (positive control, negative control, or standard) should be clearly specified.
5	Detailed explanation of methodology.	Clarity of procedure, method of standardisation, and details of any universal standards used (if applicable) should be clearly stated.
6	Operator details.	Number of operators and details regarding training and calibration of operator/s (inter-operator and intra-operator reliability) should be clearly specified.
7	Randomisation.	Details regarding sequence generation and allocation concealment should be clearly stated.
8	Method of measurement of outcome.	Clarity of procedure and rationale for choosing method should be stated. Method of standardisation along with details of any universal standards used (if applicable) should also be clearly specified.
9	Outcome assessor details.	Number of outcome assessors and details regarding training and calibration of assessor/s (inter-outcome and intra-outcome assessor reliability) should be clearly specified.
10	Blinding.	Details regarding blinding of operator(s), outcome assessor(s), and statistician should be clearly specified.
11	Statistical analysis.	Details regarding software programme used and statistical analysis should be clearly specified.
12	Presentation of results.	Outcome should be based on predefined aims and/or objectives. All data should be adequately tabulated with baseline data clearly specified (if applicable).

## Results

### Study selection

**Fig. 1:**
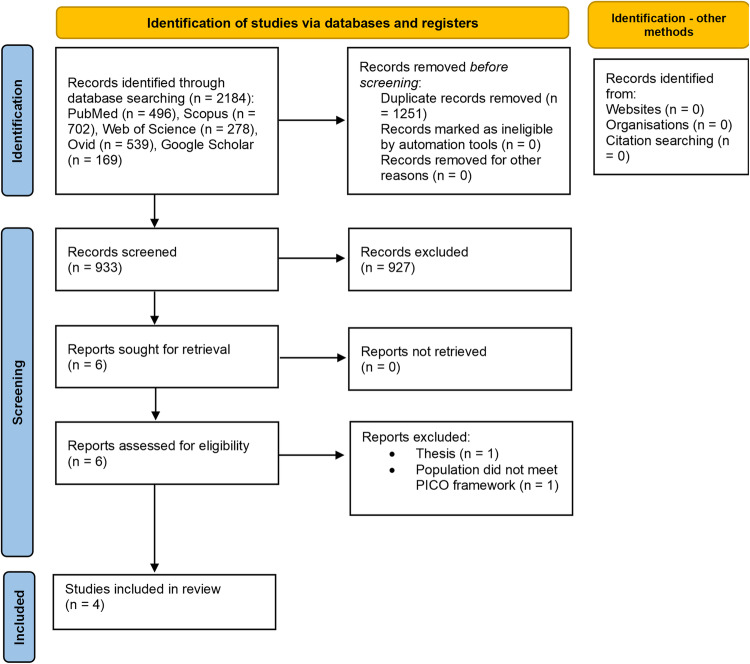
The selection process for the studies included.

2184 papers were retrieved through electronic database searching as follows: PubMed (n = 496), Scopus (n = 702), Web of Science (n = 278), Ovid Medline (n = 539), Google Scholar (n = 169). Figure 1 depicts the selection process for the studies included. The full text of 6 papers was analysed^19–24^. The paper by Plutzer was excluded because it was a thesis^19^. Additionally, Plutzer et al.^20^ published a study in 2018 with the same methodology, which was included. The study by Govindaraju et al.^21^ was also excluded because it did not use human or bovine dentine slices, as stated in the PICO framework. Manual searching did not retrieve any relevant studies. Therefore, four articles were included in this systematic review.

### Background characteristics of the included studies

The four articles included in this systematic review were in vitro and ex-vivo studies^20,22–24^. All articles were peer-reviewed and published in medical or dental journals. The studies were conducted in Australia, Thailand, Turkey and India and published between 2012 and 2021^20,22–24^. Leelapornpisid et al.^22^, Bolla et al.^23^ and Paul et al.^24^ used between 50 and 100 human-extracted teeth in the form of dentine blocks or shavings. Plutzer et al.^20^ used dentine slices from human-extracted teeth but did not specify the sample size.

The authors used a variety of test agents and microorganisms. All studies used Odontopaste as a test agent and saline or no medicament as a control^20,22–24^. Additional test agents include D,L-2-hydroxyisocaproic acid (HICA), alpha-mangostin, calcium hydroxide, Ledermix, Pulpdent, triple antibiotic paste (TAP), virgin coconut oil, sodium hypochlorite (NaOCl), propolis and chlorhexidine^20,22–24^. Additionally, one study used a combination of Odontopaste/calcium hydroxide and Ledermix/calcium hydroxide as a test agent^20^. The microorganisms used include *C. albicans*, *E. faecalis*, *L. rhamnosus* and *S. gordonii*^20,22–24^.

All studies evaluated or compared the antimicrobial capacity of various root canal medicaments^20,22–24^. Methodologies include a continuous flow cell model to grow biofilm on dentine slices^20^. Other studies used time-kill assays and the pour plate method^22,23^. Plutzer et al.^20^ used serial plating to measure microbial viability and the number of colony-forming units (CFU). Biofilm was also assessed using scanning electron microscopy (SEM)^20^. Leelapornpisid et al.^22^ used propidium monoazide quantitative polymerase chain reaction (PMA-qPCR) to evaluate the biofilm composition and the number of surviving cells. Spectrophotometry and agar culture were also used to determine cell growth^22^. Bolla et al.^23^ and Paul et al.^24^ assessed microbial capacity by measuring the number of CFU. Tests used to detect the statistical significance include the one-way ANOVA, Kruskal-Wallis, Wilcoxon signed-rank test, Pearson chi-square, Mann-Whitney and Cochran and McNemar’s test^20,22–24^. SI Table 1 summarises the included studies that evaluated the capacity of Odontopaste to reduce the microbial load in dentine.

### Qualitative review of included studies

All studies assessed the antibacterial or antifungal activity of Odontopaste compared to other medicaments^20,22–24^. One study assessed antibacterial and antifungal activity in inner dentine, deep dentine and residual root samples^22^. Another study assessed antifungal activity in dentinal shavings, one assessed antibacterial activity in dentine slices, and one assessed antibacterial and antifungal activity in dentinal shavings^20,23,24^.

When assessing antibacterial and antifungal activity, Odontopaste had significantly more microbial cell growth on roots in all dentine depths compared to HICA and alpha-mangostin^22^. Spectrophotometric analysis showed that Odontopaste was the most effective agent in inner dentine and residual roots^22^. However, calcium hydroxide and alpha-mangostin were more effective in deeper dentine^22^. Another study that assessed antibacterial and antifungal activity found that Odontopaste significantly decreased CFU compared to propolis and chlorhexidine^21^.

When assessing antifungal activity only, Odontopaste was significantly more effective than calcium hydroxide, Pulpdent, TAP and virgin coconut oil^24^. When evaluating antibacterial activity, it was concluded that Odontopaste only significantly decreased microbial numbers when compared to Ledermix^20^. The calcium hydroxide/Odontopaste combination effectively reduced microbial viability^20^. However, adding Odontopaste did not significantly enhance the effectiveness of calcium hydroxide^20^.

### Risk of bias

The studies by Plutzer et al.^20^, Bolla et al.^23^ and Paul et al.^24^ had a high risk of bias, whilst the study by Leelapornpisid et al.^22^ had a medium risk of bias. No studies explained the sample size calculation^20,22–24^. Only the study by Leelapornpisid et al.^22^ had details of a predefined population. The studies by Bolla et al.^23^ and Paul et al.^24^ specified inclusion criteria but failed to specify a predefined population or exclusion criteria. Plutzer et al.^20^ did not mention a predefined population, inclusion, or exclusion criteria. Operator details, outcome assessor details and blinding were not mentioned or conducted in any of the studies^20,22–24^. Only Leelapornpisid et al.^22^ mentioned randomisation and provided adequate information on the measurement method. Table 3 shows the scoring and categories selected for each study.

**Table 3:** Risk of bias assessment using the QUIN Tool.

Study	Aims and Objectives	Sample Size	Sampling Technique	Comparison Group	Methodology	Operator Details	Randomisation	Outcome Measures	Outcomes Assessor Details	Blinding	Statistical Analysis	Results	Total Score	Final Score	Risk of Bias
Leelapornpisid et al.^22^	2	0	2	2	2	0	1	2	0	0	2	2	15	62.50%	MEDIUM
Paul et al.^24^	2	0	1	1	2	0	0	1	0	0	2	2	11	45.84%	HIGH
Plutzer et al.^20^	2	0	0	1	2	0	0	1	0	0	2	2	10	41.66%	HIGH
Bolla et al.^23^	2	0	1	2	2	0	0	1	0	0	1	2	11	45.84%	HIGH

## Discussion

This systematic review has identified limited evidence regarding the potential benefits of Odontopaste compared to other intracanal dressings. The studies conducted by Bolla et al.^23^ and Paul et al.^24^ determined that Odontopaste had greater antimicrobial efficacy compared to calcium hydroxide and TAP, and propolis and chlorhexidine, respectively. This can be attributed to chlorhexidine’s inability to create a physical barrier against microbes, increasing the risk of root canal system reinfection when it is used as an intracanal medicament^25^. Plutzer et al.^20^ and Leelapornpisid et al.^22^ stated that Odontopaste was less effective than calcium hydroxide. In addition, Australian Dental Manufacturing has advised that Odontopaste should not be mixed with an excess of calcium hydroxide as it may compromise the steroid component of the medicament^12^.

The papers by Plutzer et al.^20^ and Leelapornpisid et al.^22^ emphasised biofilms as a more accurate experimental representation than planktonic cells of the root canal environment. The literature substantiates that cells within biofilms display a heightened resistance to antimicrobial agents, estimated to be 1000 times greater than their counterparts growing in a planktonic manner^26^. Plutzer et al.,^20^ Bolla et al.^23^ and Paul et al.^24^ tested singular microbes not representative of true endodontic biofilms. Leelapornpisid et al.^22^ performed two experiments: an in-vitro time-kill assay of planktonic organisms and an ex-vivo tooth model with a multispecies biofilm. The time-kill assay has limited clinical relevance as it tests microbes in their planktonic state. Paul et al.^24^ also used an *ex-vivo* tooth model with a single microbe. Plutzer et al.^20^ examined mature single-species biofilms extracted from human dentine slices in a continuous flow cell. Bolla et al.^23^ used a pour plate method. Unlike the other studies, they did not introduce additional cultured microbes^23^. Instead, the medicaments were applied to extracted nonsterile teeth and the resultant bacterial inoculum^23^. The medicaments were not tested against whole dentine and biofilm, but against a less structured planktonic form, making this the least representative method of the root canal system discussed^23^.

Leelapornpisid et al.^22^ labelled Odontopaste as a slow-killing agent. Plutzer et al.^20^ found that Odontopaste had no statistically significant effect, although it did eliminate 71.1% of bacteria. It is possible that Plutzer et al.^20^ found the antimicrobial reduction to be lower than the other studies because of the difference in microbial incubation time. This study determined that four weeks were needed to achieve complete biofilm structural maturity, which was more than the other studies^20^. This suggests the other studies were testing immature biofilm ecosystems, which could be more susceptible to intracanal medicaments, potentially affecting the results^22–24^. Ledermix and Odontopaste were found to be equivalent by Paul et al.^24^, however, Plutzer et al.^20^ found that Odontopaste was more effective. The critical difference between Odontopaste and Ledermix is their antibiotic ingredient, clindamycin hydrochloride and demeclocycline hydrochloride, respectively. These components are considered to have equivalent antibacterial efficacy^13^.

In a study focusing on intracanal medicaments in dentistry, the selection of microorganisms is likely driven by their relevance to dental infections and their prevalence in endodontic cases. Each microorganism can serve a specific purpose in evaluating the effectiveness of a medicament. *Candida albicans*, a prevalent fungus in the oral cavity, is included to gauge the medicaments’ performance against fungal infections, even though it may not be the primary pathogen in endodontic cases but can be present in patients with oral candidiasis^27^. *Enterococcus faecalis*, a resilient Gram-positive bacterium frequently associated with persistent endodontic infections, can be used to assess medicament efficacy in addressing challenging infections within the root canal system^27^.

Additionally, *Streptococcus gordonii*, a species commonly present in dental plaque and representing early colonisers of the dental biofilm, is incorporated to assess the medicament’s effectiveness against these specific types of bacteria^28^. By including this diverse array of microorganisms associated with endodontic infections, researchers aim to simulate the intricate microbial environment found in infected root canals in situ, facilitating a comprehensive assessment of intracanal medicaments’ broad-spectrum efficacy against various microorganisms involved in dental infections.

Two common pathogenic microorganisms that may be involved in endodontic infections are the bacterium *Enterococcus faecalis* and the fungus *Candida albicans*^27^. It has been shown that these microorganisms can resist alkalinity provided by calcium hydroxide^29–31^. All four studies in this review tested at least one of these microbes with the chosen medicaments^20,22–24^. Leelapornpisid et al.^22^, Bolla et al.^23^, and Paul et al.^24^ compared Odontopaste to other medicaments against *C. albicans*. Leelapornpisid et al.^22^ concluded that Odontopaste is ineffective in preventing *C. albicans* infections, suggesting that the medication’s steroid component may promote heightened growth. The study by Bolla et al.^23^ did not explicitly discuss Odontopaste’s effect on *C. albicans*; however, the results show no antifungal effect. On the other hand, Paul et al.^24^ claimed that Odontopaste achieved good antifungal action against *C. albicans*. Thus, further studies are needed to assess the efficacy of Odontopaste against this microorganism.

Leelapornpisid et al.^22^, Plutzer et al.^20^, and Bolla et al.^23^ compared Odontopaste to other medicaments against *E. faecalis*. The study by Leelapornpisid et al.^22^ observed a multispecies biofilm and concluded that Odontopaste is a slow-killing agent, with small microbial numbers remaining after seven days of medicament exposure. When considering this observation, it is essential to note that Odontopaste is a bacteriostatic agent rather than bactericidal^14^. Therefore, incomplete elimination should not be considered a treatment failure^14^. Bolla et al.^23^ found that Odontopaste had the most remarkable antimicrobial efficacy compared to chlorhexidine and propolis. On the contrary, Plutzer et al.^20^ pointed out that Odontopaste did not have a significant effect on *E. faecalis*. Plutzer et al.^20^ argued that clindamycin in Odontopaste is ineffective against *E. faecalis* due to the bacterium’s intrinsic resistance. It suggests that Odontopaste’s action may be due to its anti-inflammatory steroid component. However, Bolla et al.^23^ stated that Odontopaste has a sufficiently high concentration as a topical medicament (50,000 micrograms per mL) to overcome this resistance. It’s important to note that there are limitations to the effectiveness of calcium hydroxide on the steroid and antibiotic components of Odontopaste. However, using D-amino acids has shown to be beneficial in reducing *E. faecalis* biofilms when used in conjunction with Odontopaste^32^.

This systematic review has found that only the study conducted by Leelapornpisid et al.^22^ had negative controls. In this study, saline was a suitable negative control because both bacteria and *Candida* grew after 7 days of incubation^22^. However, no positive controls or inclusion/ exclusion criteria were included in the text^22^. Positive and negative controls are integral components of experimental design, serving as benchmarks to validate the reliability and sensitivity of the experimental setup. These controls enhance the robustness of experimental results by establishing a baseline for comparison and ensuring that observed effects are attributable to the experimental variables rather than methodological flaws.

Inclusion and exclusion criteria play a pivotal role in defining the parameters of a study and ensuring its relevance and validity. Inclusion criteria outline the characteristics that subjects must possess to be eligible for participation, while exclusion criteria identify factors that would disqualify individuals. These criteria help researchers precisely target their study population, reducing confounding variables and enhancing the internal validity of the study. By clearly defining the parameters of inclusion and exclusion, researchers can ensure that the study’s findings are more generalisable, reliable, and applicable to the specific population of interest. Hence, not including these inclusion and exclusion criteria, is another major limitation and can be improved and included in future studies for Odontopaste.

It should be noted that there are limitations to this systematic review. Firstly, there are limited moderate-quality research papers on the use of Odontopaste. This limits the ability of the systematic review to conclude the microbial effectiveness of Odontopaste. Secondly, the selected studies did not reach a consensus due to different methodological approaches^20,22–24^. Thirdly, each paper was found to have a medium to high risk of bias^20,22–24^.

The selected studies employed diverse methodological approaches, with variations in the use of planktonic cells and simple biofilms, as well as discrepancies in the duration of microbial exposure to individual medicaments. Notably, Plutzer et al.^20^ and Leelapornpisid et al.^22^ underscored the significance of biofilms as a more accurate representation of endodontic conditions. However, the studies by Bolla et al.^23^ and Paul et al.^24^ were conducted in vitro, precluding the creation of biofilms with multiple bacterial species. Another noteworthy limitation is the absence of specific information in Plutzer et al.^20^ and Leelapornpisid et al.^22^ regarding the reasons for tooth extraction, thereby lacking clarity on whether the infected root canals resulted from primary or persistent infections. Consequently, this uncertainty introduces variability in bacterial loads and potentially diverse bacterial species within the samples. Collectively, these methodological and diagnostic limitations hinder the ability to derive consistent conclusions from the studies included in this systematic review.

Further research is crucial to better understanding Odontopaste and optimising its use in endodontics. To achieve this, it is recommended to identify the best technique for applying Odontopaste, compare its effectiveness against biofilms instead of monoculture bacteria, conduct long-term clinical trials, examine the relationship between Odontopaste and postoperative pain, and study its biocompatibility and tissue response.

## Conclusion

Insufficient high-quality evidence exists to draw a robust conclusion on the microbial effectiveness of Odontopaste compared to other endodontic medicaments. Therefore, it is necessary to conduct additional high-quality studies of a similar nature with a low risk of bias to establish Odontopaste’s antimicrobial efficacy and whether its use will improve endodontic treatment outcomes.

## Supplementary information

Supplementary Information

## Data Availability

The data that support the findings of this systematic review are available on request from the corresponding author.
