# Supplementary material for: Antimicrobial efficacy of Odontopaste in endodontics: a systematic review
Source: Evid Based Dent. 2024 Mar 27;25(3):166. doi: 10.1038/s41432-024-01000-y (PMC11436364; doi:10.1038/s41432-024-01000-y)
Supplement: Supplementary file 1 — Supplementary Information [file 41432_2024_1000_MOESM1_ESM.pdf]

Supplementary Materials

Supplementary Table S1. Data extraction of the included studies

**Author (Year)**	**Type of study**	**Sample characteristics**	**Sample size**	**Preparation of samples**	**Microorganisms**	**Test agents and control**	**Main outcomes**
Leelapornpisid et al., 2021	In-vitro	Human teeth with a single root canal, no fractures or root curvatures. Additionally, teeth with an oval root canal shape were not selected.	100	The teeth were autoclaved. Bone, calculus and soft tissue were removed from the external root surface. The teeth were cut into root dentine blocks 6mm long and 4mm wide.<br><br>17% (w/v) Ethylenediaminetetraacetic acid (EDTA) and 0.5% (v/v) NaOCl were used for 5 minutes to remove the smear layer. The blocks were then autoclaved at 121°C for 30 minutes in 3mL of brain heart infusion (BHI) broth.<br><br>The microorganisms were exposed to the test agent for 7 days and samples	The root canals were filled with a suspension each containing 1.5 x 10^8 CFU/mL of *C. albicans, E. faecalis, L. rhamnosus, S. gordonii*. The root blocks were cultured at 37°C for 21 days.	<u>Test agents:</u><br>- HICA (40% w/v) n = 20.<br>- Alpha-mangostin (1% w/v) n = 20.<br>- Calcicur (calcium hydroxide 45% w/v) n = 20.<br>- Odontopaste n = 20.<br><br><u>Negative control:</u><br>- Sterile saline (0.9% w/v) n =20.	<u>Viability for bacterial/fungal activity:</u><br>- Following exposure to Calcicur and Odontopaste, significantly more roots displayed microbial cell growth compared to alpha-mangostin and HICA in all dentine depths (p<0.0001).<br><br><u>Spectrophotometric analysis for bacterial/fungal inhibition:</u><br>- In inner dentine and residual roots, Odontopaste was the most active agent against bacterial/fungal growth. However, in deeper dentine,

				were taken from inner dentine, deep dentine and residual roots.			Calcicur and alpha-mangostin were more effective compared to Odontopaste.<br><br><u>PMA-qPCR analysis:</u><br>- HICA and alpha-mangostin significantly reduced live cells and colony-forming equivalents (CFE) compared to Calcicur and Odontopaste ($p<0.05$).<br>- Alpha-mangostin and HICA decreased CFE by 99.9% in deeper dentine. This was higher than Odontopaste and Calcicur ($p<0.0001$).<br><br><u>Time-kill assays:</u><br>- All planktonic microorganisms were killed by Calcicur and HICA within 24 hours. However, 10 planktonic

							organisms remained after Odontopaste exposure for 7 days.<br><br>- After 7 days, alpha-mangostin, Calcicur and HICA had a significant effect against bacterial isolates and *C. albicans* compared with the control ($p<0.0001$). This was shown by a growth reduction of 5–7-Log. In comparison, Odontopaste had a 3–5-Log reduction compared to the control ($p<0.0001$).
Paul et al., 2020	In-vitro	Anterior mandibular and maxillary permanent human extracted teeth were used. Additionally, only teeth with complete root formation were selected.	70	The teeth were sectioned to create dentine blocks. The smear layer was then removed with 5.25% NaOCl and 17% EDTA for one minute.<br><br>The dentine blocks were autoclaved for 20 minutes at 121°C. The dentinal blocks were then inoculated with *C.*	*C. albicans*<br><br>- After inoculation, the dentinal blocks were incubated for 21 days at 37°C and C. albicans was renewed every 3rd day.	Test agents:<br><br>- Group I: calcium hydroxide n = 10.<br><br>- Group II: Odontopaste n= 10.<br><br>- Group III: Ledermix n= 10.<br><br>- Group IV: Pulpdent n = 10.	- After days 1 and 7, Odontopaste and Ledermix were most effective against *C. albicans*. This was statistically significant compared to the four other medicaments ($p<0.01$).

				albicans and medicaments were placed in the canal. Specimens of dentinal shavings were collected after day 1 and after day 7.		- Group V: tri-antibiotic paste n = 10.<br>- Group VI: virgin coconut oil n = 10.<br><br>Control:<br>- Normal saline n = 10.<br><br>Each group was then subdivided into day 1 and day 7.	
Plutzer et al., 2017	In-vitro	Human extracted teeth.	Not stated.	Extracted teeth were sectioned and decoronated to produce dentine slices. The smear layer was then removed with 17% w/v EDTA and 1% v/v NaOCl for 4 minutes.<br><br>The dentine samples were exposed to the medicaments for 24 hours or 48 hours. Regarding, NaOCl, the samples were placed in NaOCl for 1, 10, 30 or 60 minutes. This	*E. faecalis*<br>- Tetracycline-resistant strain (51299 American Type Culture Collection (ATCC)).<br>- Tetracycline-sensitive strain (29212 ATCC).<br><br>A continuous flow cell model was used for	Test agents:<br>- NaOCl<br>- Ledermix<br>- Odontopaste<br>- Calxyl (calcium hydroxide)<br>- Ledermix/calcium hydroxide<br>- Odontopaste/calcium hydroxide<br>- Chlorhexidine gel 0.2%	Ledermix and Odontopaste:<br>- SEM analysis of the dentine surfaces showed an undisturbed biofilm following exposure to Odontopaste and Ledermix.<br>- When Ledermix or Odontopaste were used in isolation for 48 hours, they did not significantly decrease the microbial numbers of *E. faecalis*.

				was performed in duplicate.	biofilm growth which was achieved over 4 weeks.	Control:<br>- Phosphate-buffered saline	- However, the difference in microbial capacity between Odontopaste and Ledermix was statistically significant ($p<0.05$).<br><br>Calcium hydroxide and calcium hydroxide combinations:<br>- 24 hours and 48 hours of exposure to calcium hydroxide, calcium hydroxide/Ledermix and calcium hydroxide/Odontopaste reduced microbial viability by over 99.9%. However, the antimicrobial capacity of calcium hydroxide was not significantly increased by adding Odontopaste or Ledermix.
Bolla et al., 2012	In-vitro	Human single-rooted extracted teeth.	50	Pour plate method:<br>- Access openings were created and then 18% EDTA and 5.25% NaOCl were used.	*E. faecalis* and *C. albicans*.	Test agents:<br>- Odontopaste<br>-Propolis	The average number of bacterial colonies remaining after exposure to each agent:

				- After 24 hours of the root canal system being exposed to the medications, dentinal shavings were collected and inoculated in a BHI broth.<br><br>- 100mg of each agent was added to 5 conical flasks.<br><br>- 0.01mL of bacterial and fungal inoculum were added to the flasks.<br><br>- The mixture was poured into Petri dishes and solidified for 24-48 hours.		- Chlorhexidine (positive control)<br><br><u>Negative control:</u><br><br>- No medicament	- Odontopaste – 25700 CFU<br><br>- Propolis – 106000 CFU<br><br>- Chlorhexidine – 223000 CFU<br><br>- Negative control – 878000 CFU<br><br><u>The average number of fungal colonies remaining after exposure to each agent:</u><br><br>- Odontopaste – 11000 CFU<br><br>- Propolis – 563000 CFU<br><br>- Chlorhexidine – 398000 CFU<br><br>- Negative control – 783000 CFU<br><br>Odontopaste had a significantly lower number of CFU compared to propolis

							and chlorhexidine ($p<0.05$).

Supplementary Table S2 – Reasons for excluding articles after evaluating eligibility

	Author	Year	Title	Reason for Exclusion
1	Plutzer, B.	2009	Comparative efficacy of endodontic medicaments against *Enterococcus faecalis* biofilms.	This paper is a thesis. Plutzer published a study based on this thesis in 2018 which was included instead.
2	Govindaraju, L.	2021	Antibacterial Activity of Various Intracanal Medicament against *Enterococcus faecalis*, *Streptococcus mutans* and *Staphylococcus aureus*: An In vitro Study.	This paper did not use human or bovine dentine slices. Therefore, it did not meet the PICO framework established.
